# Spatial memory deficits in a mouse model of late-onset Alzheimer’s disease are caused by zinc supplementation and correlate with amyloid-beta levels

**DOI:** 10.3389/fnagi.2014.00174

**Published:** 2014-10-22

**Authors:** Jane M. Flinn, P. Lorenzo Bozzelli, Paul A. Adlard, Angela M. Railey

**Affiliations:** ^1^Department of Psychology, George Mason UniversityFairfax, VA, USA; ^2^Synaptic Neurobiology Laboratory, The Florey Institute of Neuroscience and Mental Health, The University of MelbourneParkville, VIC, Australia

**Keywords:** apolipoprotein E, hAPP, CRND8, transgenic mice, Barnes maze, metals, ZnT3, copper

## Abstract

Much of the research in Alzheimer’s disease (AD) that uses mouse models focuses on the early-onset form of the disease, which accounts for less than 5% of cases. In contrast, this study used a late-onset AD model to examine the interaction between increased dietary zinc (Zn) and the apolipoprotein E (ApoE) gene. ApoE ε4 is overrepresented in late-onset AD and enhances Zn binding to amyloid-β (Aβ). This study sought to determine if elevated dietary Zn would impair spatial memory in CRND8 mice (CRND8), as well as mice who carry both the mutated human amyloid precursor protein (APP) and ApoE ε4 genes (CRND8/E4). Mice were provided with either lab tap water or water enhanced with 10 ppm Zn (ZnCO_3_) for 4 months. At 6 months of age, spatial memory was measured by the Barnes maze. CRND8 mice exhibited significant memory deficits compared to WT mice, as shown by an increased latency to reach the escape box. For the CRND8/E4, but not the CRND8 mice, those given Zn water made significantly more errors than those on lab water. During the probe trial for the WT group, those on Zn water spent significantly less time in the target quadrant than those on lab water. These data suggest that increased dietary Zn can significantly impair spatial memory in CRND8/E4. WT mice given Zn water were also impaired on the 24-h probe trial when compared to lab water WTs. Within the CRND8/E4 group only, levels of soluble Aβ were significantly correlated with average primary latencies. Within the Zn-treated CRND8/E4 group, there was a significant correlation between insoluble Aβ and average primary errors. Levels of the zinc transporter 3, ZnT3, were negatively correlated with soluble Aβ (*p* < 0.01). These findings are particularly relevant because increased intake of dietary supplements, such as Zn, are common in the elderly—a population already at risk for AD. Given the effects observed in the CRND8/E4 mice, ApoE status should be taken into consideration when evaluating the efficacy of therapies targeting metals.

## Introduction

Alzheimer’s disease (AD) is the primary cause of dementia in the elderly and currently affects more than five million Americans (Alzheimer’s Association, 2013). The present research focuses on how the biometals, such as zinc (Zn), copper (Cu), and iron (Fe), interact with amyloid beta (Aβ)—the key constituent of the plaques that are characteristic of the disease, which mediates plaque formation and consequent oxidative damage.

Zn, Cu, and Fe are found in high concentrations in and around amyloid plaques (Lovell et al., 1998; Maynard et al., 2005), consistent with the notion that Aβ is a metalloprotein that possesses binding sites for both Zn and Cu (Bush et al., 1993; Hesse et al., 1994). Zn has been shown to be particularly effective in promoting Aβ aggregation (Bush et al., 1993), and the sequestration of Zn by Aβ leads to an intracellular deficiency of this metal (Grabrucker et al., 2011). Cu, on the other hand, causes a shift in the processing of the amyloid precursor protein (APP) towards the non-amyloidogenic pathway (Borchard et al., 1999) but once Aβ is present, it binds both Cu and Fe. Therefore, Cu and Fe binding is problematic as these metals are redox-active and Aβ reduces both metals, resulting in the production of hydrogen peroxide (H_2_O_2_; Huang et al., 1999). H_2_O_2_ further reacts with the reduced metal to produce hydroxyl radicals that can cause oxidative damage (Bush, 2003). Both aging and plaque-related inflammation may promote a more acidic brain environment, which is conducive to Fe and/or Cu binding (Atwood et al., 1998).

Evidence suggests that Zn is crucial for Aβ deposition, and the presence of Zn *in vitro* prevents the proteolytic degradation of Aβ_1–42_ by matrix metalloprotease 2 (Crouch et al., 2011). Zn also inhibits the activity of alpha-secretase and promotes gamma- and beta-secretase activity (Capasso et al., 2005). The zinc transporter, ZnT3, is responsible for loading zinc into the synaptic vesicles (Palmiter et al., 1996; Cole et al., 1999; Linkous et al., 2008). Eliminating synaptic Zn by genetic ablation of zinc transporter 3 (ZnT3) in an AD mouse model resulted in a 50% reduction in amyloid (Lee et al., 2002). ZnT3 levels, however, have been shown to decrease as a function of age and AD (Adlard et al., 2010). Interestingly, Bjorklund et al. (2012) found that in brains exhibiting AD neuropathology, ZnT3 levels were lowest in those patients who received a diagnosis of AD, while those who did not receive a diagnosis had ZnT3 levels similar to those of healthy controls.

Given the complexity of biometals in AD, the behavioral outcome due to changes in metal levels is difficult to predict, but this has been studied in various mouse models. Long-term dietary Zn enhancement in transgenic (Tg) mouse models of AD (TgCRND8, Tg2576, and APP/presenilin 1 (PS1) mouse models) has been shown to result in cognitive deficits (Linkous et al., 2009; Railey et al., 2011), as well as an increase in both brain Zn levels and the number and size of Zn-positive plaques in the cortex and hippocampus (Wang et al., 2010). Other findings have found that a severe Zn deficiency (<10 ppm Zn) enlarged amyloid plaques (Stoltenberg et al., 2007); thus either an overabundance or deficiency in Zn can potentiate amyloid deposition.

The observations derived from these early-onset mouse models may be of diminished utility for the majority of AD cases that are classified as late-onset (manifest after age 65 years) (Harvey et al., 2003). The emphasis on early-onset genetics dominating AD research is due to the strong relationship between those genes and development of the disease. Genetic linkages to the late-onset form of AD are less specific, but the ε4 allele of the apolipoprotein E (ApoE) gene has been identified as a major risk factor (Saunders et al., 1993; Blacker et al., 1998; Huang and Mucke, 2012). The ApoE gene has three alleles (ε2, ε3, and ε4) resulting in three isoforms of the protein (Zannis et al., 1981), with ApoE ε4 overrepresented in patients with late-onset AD (Weisgraber and Mahley, 1996). While ApoE ε4 is considered a susceptibility factor it does not guarantee the development of disease (Weisgraber and Mahley, 1996). The exact mode of action of ApoE ε4 in AD remains elusive. One proposed mechanism is the interaction between ApoE ε4 and Aβ, which may increase plaques and/or impede Aβ clearance (Mahley et al., 2006). Amongst those with AD, ApoE ε4 carriers typically have a larger number of plaques than ApoE ε3 carriers (Schmechel et al., 1993). Similarly, ApoE ε4 mice that overexpress human APP have an elevation in Aβ plaque deposition, reduced numbers of presynaptic terminals, and an impairment in learning and memory tasks as compared to their ε3 counterparts (Huang, 2006).

The ApoE alleles differ at two residues: ε2 (Cys^112^, Cys^158^), ε3 (Cys^112^, Arg^158^), and ε4 (Arg^112^, Arg^158^) (Rebeck et al., 2002). Cysteine binds to Zn and Cu with high affinity and the Zn binding site on APP is located in a cysteine-rich area (Bush and Tanzi, 2002). The increased number of cysteine residues in ε2 and ε3 correlates with their ability to bind zinc in the synaptic cleft and may contribute to the protective effect of these alleles by preventing zinc binding to Aβ (Lee et al., 2010). ApoE ε4 is also implicated in the formation of neurofibrillary tangles (NFTs). It may increase the phosphorylation of the tau protein (Harris et al., 2004) and Zn may be a key modulator of this process (Craddock et al., 2012). Through their relatively high-binding affinity for zinc, Aβ plaques will cause a reduction in intracellular levels of zinc that will in turn lead to a destabilization of microtubules and subsequent liberation of tau (Craddock et al., 2012). These findings suggest that Zn and ApoE ε4 may act together to affect multiple processes in AD through the promotion of NFT formation and increased Aβ deposition, which may be due to increased Zn binding in the presence of ApoE ε4.

In order to examine the effect of excess zinc in the more prevalent form of AD we have developed a late-onset model, examining two types of Tg mice with the (1) humanized ApoE ε4 and mutated hAPP cross (CRND8/E4); or (2) those only with mutated hAPP (CRND8), and investigated the effect of Zn supplementation on spatial memory in these mice. Protein levels of soluble and insoluble Aβ, and ZnT3 were measured to elucidate molecular mechanisms that may be associated with excess dietary zinc in AD.

## Materials and methods

### Subjects

To address the primary research questions of the study, the creation of a mouse strain modeling late-onset AD was necessary. The CRND8/E4 experimental animals were obtained by breeding CRND8 males (University of Toronto) with female mice who were homozygous for the human ApoE ε4 knock-in and the knock-out of murine ApoE (Jackson Labs). Resultant CRND8/E4 were therefore heterozygous for human ApoE ε4 and murine ApoE in addition to being hemizygous for the hAPP mutation. Tg CRND8 mice carry a mutant form of APP 695 containing both the Swedish (KM670/671NL) and Indiana (V717F) mutations on a hybrid C3H/B6 genetic background and exhibit extensive amyloid deposition by 3 months of age. There is a potent increase in Aβ_42_ at around 10 weeks of age in this model making the Aβ_42_ to Aβ_40_ ratio 5:1 (Chishti et al., 2001). The Tg1HolApoe^*tm*1*unc*^/J strain expresses human ApoE ε4 under the direction of human glial fibrillary acidic protein (GFAP). Experimental WT mice were those littermates who did not inherit the hAPP transgene from the cross between CRND8 males and WT females (C3H/B6). CRND8 mice were those offspring who inherited the hAPP transgene from the CRND8 males that were bred with WT females.

Mice were bred in three groups in order to facilitate behavioral testing. The offspring were group housed with same-sex littermates with 2–4 animals per cage. Each cage contained an igloo, nylabone, and running wheel (Bio-serv). The mouse colony was maintained with a 12-h light/dark cycle. Food (Harlan diet 7012) and water were provided *ad libidum*.

Behavioral group numbers are shown in Table 1. This study used both male and female mice, and analyses were conducted to determine possible sex differences. Zn supplementation began at 6 weeks of age and continued throughout behavioral testing. Behavioral testing began at 5 months of age and mice were sacrificed within 10 days following the conclusion of all behavioral testing (approximately 6 months of age). Mice were euthanized by CO_2_ asphyxiation and brains were removed and stored in a −80° freezer for later analysis. There were no differences in body weight on the day of euthanasia when evaluated by water condition. All experiments and procedures performed on these mice were approved by the George Mason University Institutional Animal Care and Use Committee.

**Table 1:** **Group distributions and male to female ratio for Barnes maze (BM) and Western blot (WB) analyses**.

Group	BM *N* =	BM m:f	WB *N* =	WB m:f
**WT + Lab Water**	12	7:5	0	n/a
**WT + Zn**	12	6:6	0	n/a
**CRND8/E4 + Lab Water**	11	6:5	10	5:5
**CRND8/E4 + Zn**	11	5:6	11	5:6
**CRND8 + Lab Water**	12	8:4	10	6:4
**CRND8 + Zn**	11	9:2	9	7:2

### Water preparation

The 10 ppm ZnCO_3_ was supplemented to laboratory tap water (herein referred to as Zn water) and was prepared using a starting solution of 10,000 mg/L of zinc dissolved in 5% HNO_3_. The final solution was buffered with Na_2_CO_3_ to bring it to a pH of 7.0 (Linkous et al., 2009). All waters were made and stored in separate polycarbonate carboys and dispensed to animals in 500 mL glass bottles. Water was analyzed regularly using inductively coupled plasma-optical emission spectroscopy and ion chromatography at the United States Geological Survey (USGS, Reston, VA) to confirm metal content. Laboratory tap water without any supplement, herein referred to as lab water, was used for the control group. Water consumption was measured every week and there were no significant differences when evaluated by water condition.

### Barnes maze

The Barnes maze (BM) is designed to assess spatial memory. The maze (Med Associates) consisted of an elevated platform 122 cm in diameter (91 cm above the ground) with 40 equidistant holes located around the edges. The maze’s surface color was white. An escape box was located under one of the holes and four distinctive extra-maze reference cues were placed around the maze, with one on each side (large black letters on a white background). Testing took place in a brightly illuminated room with a 300-watt light situated directly over the maze in order to make the maze surface undesirable, given the natural tendency of mice to prefer dark enclosed areas over bright/open areas. All animals started each trial from a dark enclosed start box positioned in the center of the maze. The orientation of the start chamber was varied for each trial. The maze was cleaned with 70% ethanol solution between all trials to eliminate scent trails.

#### Procedure

BM testing took place over seven consecutive days with the 24-h probe trial occurring on the seventh day.

Habituation: On the first day of testing all mice were given two habituation trials. The mice were placed in a start chamber in the center of the maze for 10 s after which time the start box was lifted and they were gently guided to the escape box and allowed to enter on their own. Once inside, mice stayed in the escape box for 30 s before being returned to their home cage for a 2 min inter-trial interval.

Acquisition: Days 2–6 were acquisition days. The mice received three trials per day with a 15 min inter-trial interval. The trials on the acquisition days ended when the animal entered the escape box or when the maximum trial length of 3 min was reached. Mice were allowed 30 s in the escape box after locating it. If the animal did not find the escape box in the allotted 3 min trial period they were gently guided to the escape box and allowed 30 s inside. Primary latency (amount of time elapsed prior to first reaching target hole) and primary errors (number of errors made prior to reaching target hole) were measured.

Probe Trial: On day 7 the animals received one probe trial 24 h after the last training trial. The escape box was removed from the target hole that had been previously learned and the mice were given 3 min to explore the maze. Percent of time in the correct quadrant was measured.

### Tissue preparation

Frozen brain samples were minced into small pieces and homogenized in 1 mL of a mixture of PBS and protease/phosphatase inhibitor cocktail (Sigma-Aldrich). The homogenate was then centrifuged at 4°C at 24,000 RPM for 1 h. Soluble and insoluble fractions were separated. The insoluble fractions were additionally reconstituted in 1 mL of a mixture of PBS and protease/phosphatase inhibitors. Both soluble and insoluble fractions were then lyophilized for approximately 12 h until fully dehydrated.

### Western blot analyses

For Western blot (WB) analysis, lyophilized insoluble fractions were reconstituted in 250 µL of PBS and protease/phosphatase inhibitor cocktail. Soluble fractions were reconstituted in 50 µL of PBS and cocktail. A BCA protein assay was done to determine protein concentration for each sample. Fifteen microliters of samples were heated at 90°C for 10 min and then spun down and loaded onto Criterion XT precast 4–12% gels. After the gels were run and transferred to a PVDF membrane they were blocked in 5% tris buffered saline with Tween20 (TBST) skim milk for 1 h. The membranes were then briefly rinsed with TBST and incubated overnight at 4°C with the following primary antibodies: WO2 (1:2000; in-house antibody prepared at The Florey Institute of Neuroscience and Mental Health); ZnT3 (1:10,000, in-house antibody prepared at The Florey Institute of Neuroscience and Mental Health); and GAPDH (1:10,000; Millipore Cat: MAB 374). After three washes of TBST for 5–10 min, secondary antibody incubation was performed (polyclonal rabbit anti-mouse IgG conjugated to horseradish peroxidase (HRP); 1:10,000; Dako P0260) at room temperature for 1 h. Three additional washes of TBST were done before enhanced chemiluminescent substrate was added for imaging using Multigauge software (Fujifilm). After imaging, the membranes were briefly rinsed with TBST and then the membranes were stripped using Re-blot Plus Strong solution (Millipore). The bands were scanned and the intensities of the bands were measured using the Fuji Reader LAS-4000. Protein levels for WO2 and ZnT3 were normalized to GAPDH (Figure 5).

**Figure 1:**
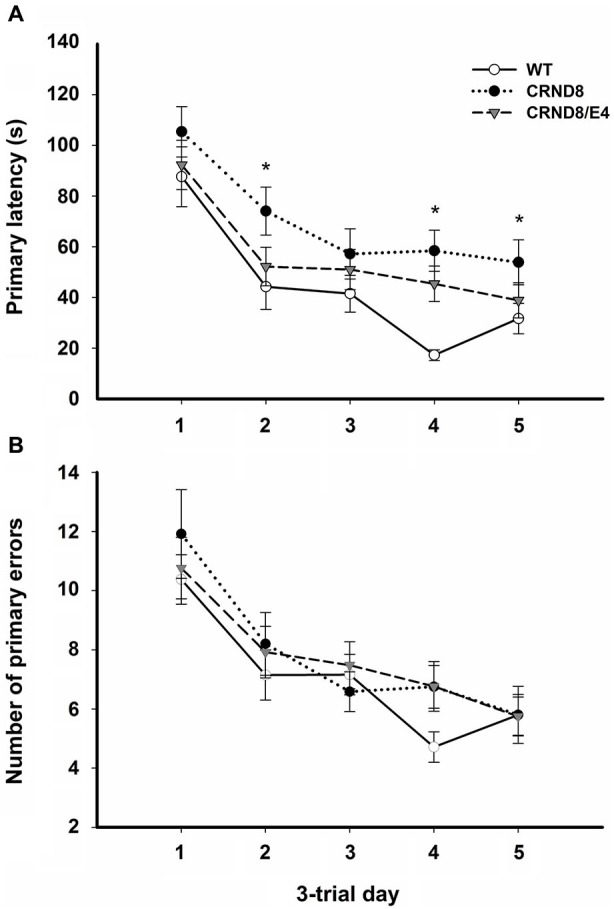
**Average primary latency and primary errors across genotypes in the Barnes maze, regardless of water condition. (A)** Average primary latencies were significantly shorter for WT mice as compared to CRND8 mice (*p* < 0.05), but not CRND8/E4s. Overall, there was a significant decrease across days in primary latency for all genotypes (*p* < 0.001). **(B)** Average primary errors in the Barnes maze. Primary errors were not significantly different between any of the genotypes. All groups did, however, improve significantly over time (*p* < 0.001). Points are mean ± SEM. * denotes *p* < 0.05.

**Figure 2:**
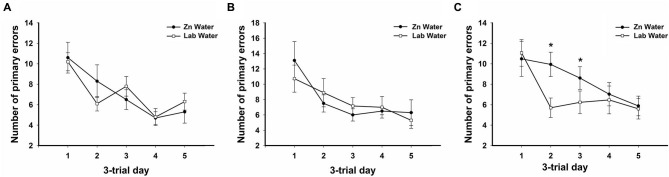
**Average primary errors for all genotypes.** There were no significant differences between Zn and Lab water **(A)** WT and **(B)** CRND8 mice. **(C)** Within the CRND8/E4 mice, however, those on Zn water had significantly more primary errors than the lab water group on days 2 and 3 (*p* < 0.05). Points are mean ± SEM. * denotes *p* < 0.05.

**Figure 3:**
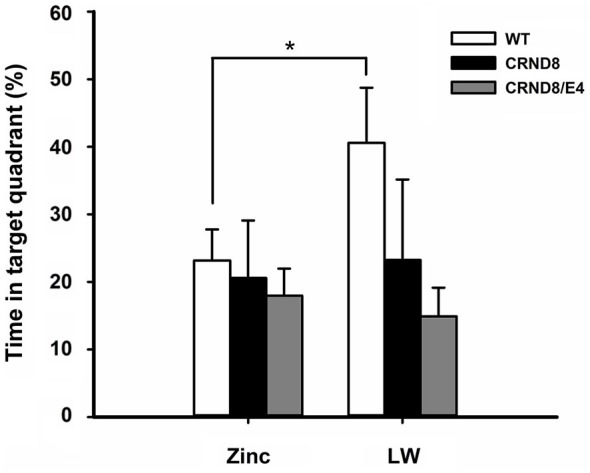
**Percent of time spent in the target quadrant in the Barnes maze.** WT mice on Zn water spent significantly less time in the target quadrant during the 24-h probe trial when compared to lab water WT mice (*p* < 0.05). Tg mice consistently scored at or below chance levels. Points are mean ± SEM. * denotes *p* < 0.05.

**Figure 4:**
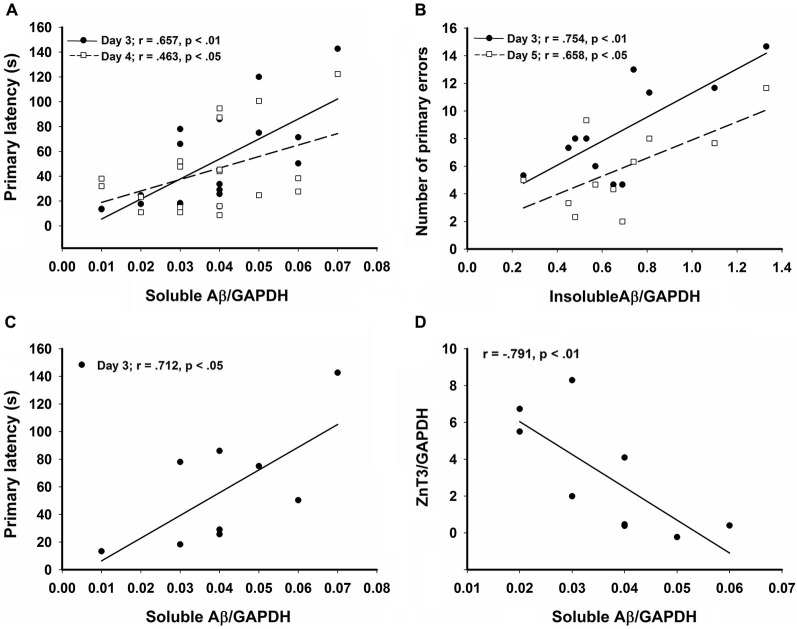
**Significant correlations in the CRND8/E4 group showing (A) soluble Aβ vs. average primary latencies for days 3 (*r* = 0.657, *p* < 0.01) and 4 (*r* = 0.463, *p* < 0.05); (B) In Zn water mice, insoluble Aβ vs. average primary errors for days 3 (*r* = 0.754, *p* < 0.01) and 5 (*r* = 0.658, *p* < 0.05); (C) In lab water mice, soluble Aβ vs. average primary latency on day 3 (*r* = 0.712, *p* < 0.05); and (D) In Zn water mice, soluble Aβ negatively correlates with ZnT3 (*r* = −0.791, *p* < 0.01).** * denotes *p* < 0.05.

**Figure 5:**
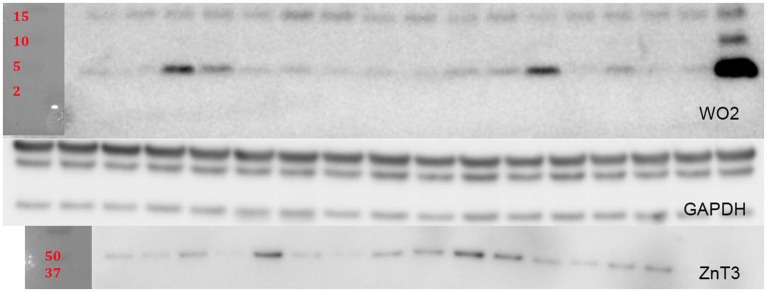
**Representative blots are shown for WO2 and ZnT3 which were normalized to GAPDH**.

### Statistical analyses

Statistical differences among different genotypes and water conditions against measured behavioral parameters were determined by repeated-measures analysis of variance (RMANOVAs). When necessary, Greenhouse-Geisser estimates of sphericity were used to correct for degrees of freedom. Bonferroni *post-hoc* tests were used to evaluate group differences. Correlations between protein levels and measured behavioral parameters were calculated using the Pearson correlation coefficient (*r*). Data are expressed as mean ± SEM. A significance level of *p* < 0.05 was used for all analyses. Trends were reported at a level of *p* < 0.10.

## Results

### Barnes maze behavior

Three genotypes (WT, CRND8/E4, CRND8) × two water conditions (lab water and zinc water) mixed design RMANOVAs were performed for measures of primary latency, primary errors, and percent of time in the target quadrant for the 24-h probe trial. Between group variables were genotype and water type. The within subjects variable was testing day (five levels). Sex was entered as a covariate for all statistical analyses.

On days 1–5, there was a significant within-subjects effect of day for primary latency (*F*_(3.03,172.43)_ = 44.29, *p* < 0.001) indicating overall improvement across testing days for all groups. There was a significant effect on primary latency between genotypes (*F*_(2,57)_ = 4.124, *p* < 0.05). A Bonferroni *post-hoc* test revealed that primary latencies across days 1–5 were significantly shorter for WT mice as compared to CRND8 mice (*p* < 0.05), but not compared to CRND8/E4. The CRND8/E4 were also not significantly different from CRND8 mice (Figure 1A). On average CRND8/E4 animals had latencies that were shorter than CRND8 animals, but longer than WT animals. For primary latency, there were no main effects of water condition.

On days 1–5, there was a significant within-subjects effect of day for primary errors (*F*_(3.39,200.0)_ = 23.32, *p* < 0.001). For primary errors there were no significant main effects of water or genotype across all groups on days 1–5 or during probe trials (Figure 1B). Further analyses within genotypes revealed no significant differences between Zn water and lab water in WT and CRND8 mice (Figures 2A,B). However, within the CRND8/E4 genotype there was a significant main effect of water. CRND8/E4 mice on Zn water made significantly more primary errors than those CRND8/E4 on lab water on days 2–3 (*F*_(1,17)_ = 5.64, *p* < 0.05; Figure 2C).

There were no significant main effects of genotype on the 24-h probe trial. There was, however, a significant main effect of water condition within the WT group. Zn water WT mice spent significantly less time than lab water WT mice in the target (correct) quadrant (23% vs. 41% respectively) (*F*_(1,21)_ = 3.28, *p* < 0.05; Figure 3). Both CRND8/E4 and CRND8 groups raised on either Zn or lab water scored close to chance during the probe trial.

In summary, there were significant main effects of water condition within the CRND8/E4 and WT genotypes, but not in the CRND8 group. As predicted, Zn water CRND8/E4 mice had higher primary errors in the BM compared to those on lab water. Significant genotype differences did exist between CRND8 mice compared to WT mice on measures of primary latency, as was expected. Mice carrying both the ApoE ε4 and mutated hAPP genes were not more impaired than the CRND8 transgene. However, on the primary error measure, CRND8/E4 mice on Zn water had the highest number of primary errors. WT mice on Zn water were spent significantly less time in the target quadrant when compared to those WTs on lab water.

### Protein analysis

Relative levels of soluble Aβ, insoluble Aβ, and ZnT3 were obtained from WB analysis. This analysis was conducted only on Tg mice (CRND8 and CRND8/E4). The control for both Tg groups was a CRND8/E4 in the lab water group which may explain why all significant correlations with behavior were seen only in the CRND8/E4 group; all protein correlations presented here are within that genotype only. There were no significant main effects for water and genotype. There were no significant main effects on levels of ZnT3. There were also no significant main effects for soluble Aβ or insoluble Aβ. Regardless of water condition, however, levels of soluble Aβ were significantly correlated with the average primary latencies for day 3 (*r* = 0.657, *p* < 0.01) and day 4 (*r* = 0.463, *p* < 0.05; Figure 4A). Those mice on Zn water had levels of insoluble Aβ that were significantly correlated with average primary errors for day 3 (*r* = 0.754, *p* < 0.01) and day 5 (*r* = 0.658, *p* < 0.05; Figure 4B). Those on control lab water had soluble Aβ levels which significantly correlated with the average primary latency on day 3 (*r* = 0.712, *p* < 0.05; Figure 4C). Mice on Zn water had levels of soluble Aβ that were negatively correlated with levels of ZnT3 (*r* = −0.791, *p* < 0.01; Figure 4D).

## Discussion

These data show that the performance of the mice modeling late-onset AD depended significantly upon the presence or absence of Zn in the drinking water. Overall, CRND8/E4 mice did not perform worse than the CRND8 mice, as had initially been predicted. However, CRND8/E4 mice raised on Zn water made the most errors in the BM of all six groups, and also had significantly increased errors compared to those raised on lab water. On the primary latency measure, the CRND8 mice performed significantly worse than the WT mice. The CRND8/E4 group’s performance was intermediate between the other groups, and did not differ significantly from either. In contrast to the effect on primary errors, there was no significant effect of Zn water on primary latency. The observation that primary errors, and not primary latencies, detected a Zn effect in the late-onset mouse model is consistent with O’Leary and Brown (2013) who have suggested that errors are a more sensitive measure of memory than latency in the Barnes maze.

The effect on errors may be due to enhanced Zn binding to Aβ when in the presence of ApoE ε4. ApoE ε4 has the weakest affinity for binding Zn, due to the amino acid substitution of arginine which is not a ligand for Zn (Lee et al., 2010). In contrast, the ApoE ε2 and ApoE ε3 isoforms with cysteine residues strongly bind Zn, and may prevent it from binding with Aβ, which could in turn prevent oligomerization and deposition (Lee et al., 2010). While there are numerous *in vitro* studies investigating the association between Zn and ApoE ε4, this is a novel behavioral study examining the relationship between the two in a mouse model of late-onset AD.

Zn water did not result in significant latency deficits in the CRND8 and WT groups. For the WT group, Zn significantly reduced the percent time spent in the target quadrant for the 24-h probe trial; thus the WT mice on Zn water were impaired on the most difficult memory task. Neither the CRND8/E4 or CRND8 groups performed above chance on either water condition (Figure 4); thus, additional impairments caused by Zn were difficult to assess. Given the difficulty of the 24-h probe task for the Tg mice, running probe tests as the last run on alternate days, as is done in the Morris water maze (MWM), could be a useful strategy.

We have previously demonstrated significant latency impairments in the MWM in both Zn water rats and Tg (CRND8 and Tg2576) mice (Linkous et al., 2009; Railey et al., 2010, 2011). The MWM is a more stressful test than the BM (Hölscher, 1999) and this may account for the different effects of zinc on latency seen in this experiment. Overall, data from our lab indicate that Zn water has adverse behavioral effects on WT and CRND8 mice but that additional mechanisms are at work for CRND8/E4 mice. Although Zn supplementation exacerbated behavioral deficits in the Tg2576 and CRND8 mice, this led to a paradoxical decrease in amyloid deposits in the Tg2576 mice (Linkous et al., 2009), which was also observed by Harris et al. (2014); however they found no significant behavioral differences.

The deficits in spatial memory that were observed in the Barnes maze, in connection with dietary Zn supplementation, may be attributable to the effect of Zn on other metals in the brain, specifically Cu. Excess dietary Zn prevents adequate absorption of Cu through the intestinal wall (Milne et al., 2001). Therefore, Zn supplementation may potentiate a Cu deficiency that is inherent in Tg mice overexpressing mutant hAPP (Phinney et al., 2003). Brewer (2014), however, found a non-significant delay in cognitive impairment in AD patients who received Zn supplements, and suggests that AD is the result of a Zn deficiency. He also noted that these subjects had relatively low Zn serum levels and suggested that this would lead to an overload of copper. Brewer (2014) considers zinc deficiency a risk factor for AD, due to its interaction with copper (Brewer, 2014).

In contrast, Klevay (2008) has suggested that AD is due to a Cu deficiency instead of Cu toxicity and spatial memory deficits in Zn-treated Tg mice can be remediated by the concurrent administration of small amounts of Cu (Railey et al., 2011). APP-overexpressing mice have lower levels of Cu in the brain (Bayer et al., 2003) which would support increasing dietary Cu to treat AD. This is consistent with another study where APP23 mice were given Cu supplementation which resulted in a reduction of Aβ and an increase in superoxide dismutase 1 (SOD1) activity (Bayer et al., 2003). These conflicting findings from different labs suggest that metal dyshomeostasis in AD can be due to either a deficiency or overabundance of metal ions, and that alterations to the levels of one metal can impact levels of another. The initial metal status of the patient should be an important factor in considering any form of dietary metal-based therapy.

In contrast to our observations, Zn supplementation in the 3xTg mouse actually improved performance on behavioral tasks, and decreased both Aβ and tau pathology (Corona et al., 2010), while decreased dietary Zn elevated plaque volume in APP/PS1 mice (Stoltenberg et al., 2007). Carrying mutations in PS1, or in the case of the 3xTg, a mutation in tau, can alter the ability of these proteins to interact with metals. The divergent results may stem from differences in the mouse model used, chemical composition of zinc solutions, and age of onset and duration of Zn supplementation. Our mice were given a standard rodent chow which contains adequate levels of Zn, in addition to the Zn supplementation, which would be in contrast to the Zn deficiency that is reported in the elderly population. While Zn therapy may be beneficial for those with a Zn deficiency, excess Zn, and Zn in the presence of ApoE ε4, may actually be harmful.

In a mouse model carrying the three different human ApoE alleles in the 5xTg mouse, the ApoE transgenes delayed pathology typically observed in the 5xTg strain (Youmans et al., 2012). Although having an expanded window of time to study the progression of AD pathology could be useful, using either the 3xTg or 5xTg strains may be contraindicated for studying the effects of Zn or other metals in late-onset AD. These multiple transgene models have mutations in PS1, which affects cellular turnover of Zn and Cu, since PS1 has Zn binding sites, which will affect SOD1 activity (Sensi et al., 2008; Greenough et al., 2011; Southon et al., 2013). Therefore, studies looking at the relationship between metals and pathobiology of late-onset AD should avoid incorporating mutated PS1 transgenes. Also, enzymes that modulate tau are Zn-dependent.

The current data did not support the predicted outcome of more behavioral impairment in CRND8/E4 mice as compared to CRND8 mice. On most measures the CRND8/E4 mice performed slightly, but not significantly, better than CRND8 mice. This is consistent with other recent behavioral data in younger Tg mice modeling late-onset AD (Moreau et al., 2013). Similar observations of intermediacy were reported in circadian rhythm and nest building behavior, as well as in markers for inflammatory cytokines (Graybeal et al., 2014). Recent studies have shown that any isoform of human ApoE delays AD pathology in early-onset Tg mice (Youmans et al., 2012; Tai et al., 2013).

One possible explanation for the greater impairment associated with the ApoE ε4 allele in old age is due to its interaction with metals and their dysregulation with increased age. Zn and Cu have an interdependent relationship and they exist in a delicate homeostasis whereby a shift towards either excess or deficiency may influence Aβ neurotoxicity. Lee et al. (2012) have shown that Zn accumulates around dystrophic neurons in aged Tg2576 mice and suggest that this excess Zn “crowded around amyloid plaques” and could directly cause the death of neurons. Such an effect would likely be greater in the case of ApoE ε4 which does not bind Zn and Cu as efficiently as the other forms of ApoE.

Another interesting finding was that ZnT3, which is the transporter responsible for loading zinc into synaptic vesicles, was negatively correlated with soluble Aβ levels, which is the more toxic form of Aβ. Although a general decline in levels of ZnT3 has been associated with cognitive decline (Bjorklund et al., 2012), it has also been demonstrated that the regions of brain that express ZnT3 may change so that the overall level of expression remains unaltered, but the localization of expression may vary and be centered more around areas of pathology in the Tg CNS (Lee et al., 2012). This may explain why no overall differences for ZnT3 expression were detected. It is notable that within the Zn water group we observed a correlation between insoluble Aβ and primary errors, as this may be due to the ability of Zn to aggregate Aβ. The relationship between amyloid load and learning and memory remains controversial; for example, Gruart et al. (2008) found no relationship between hippocampal-dependent learning and amyloid load, suggesting that other mechanisms may be involved.

ApoE ablation reduces the amount of ZnT3 and results in significantly lower levels of synaptic Zn compared to WTs (Lee et al., 2010). Zn is required for both presynaptic and postsynaptic mossy fiber long-term potentiation, and dysregulation of synaptic Zn alters the excitatory input of the mossy fiber-CA3 synapse in the hippocampus (Pan et al., 2011). Furthermore, ApoE-ε4 targeted replacement mice have altered levels of glutaminase and vesicular glutamate transporter 1 (Dumanis et al., 2013). ApoE-ε4 is correlated with decreased dendritic spines (Dumanis et al., 2009), and treatment of neurons with ApoE-ε4 recombinant protein reduces expression of glutamate receptors (Chen et al., 2010). Future research should examine the effects of Aβ, Zn supplementation, and ApoE status on glutamatergic pathways.

Since this study demonstrates that CRND8/E4 mice are sensitive to dietary Zn-enhancement, this suggests that therapies targeting metals should consider ApoE allele status in their analyses. Currently, the only FDA-approved drugs for treating AD work by alleviating symptoms instead of targeting the underlying etiology of the disease. A newer class of drugs currently under evaluation, the 8-hydroxyquinolines (8-OHQ), are selective Zn/Cu chaperones (Greenough et al., 2013). Two drugs in this class, Clioquinol (CQ) and PBT2 have a higher affinity for binding Zn and Cu when compared to Aβ. Instead of chelating these metals from the system, they are able to return metals back inside the cell, thereby restoring the bioavailability of metal ions necessary for cellular processes (Adlard and Bush, 2012). This restoration of intracellular metal concentrations has been shown to increase synaptic proteins, reduce Aβ accumulation, and improve cognitive functioning in Tg mice (Adlard et al., 2008, 2011; Crouch et al., 2011).

## Conclusions

This experiment showed that Zn water caused significant impairments in a mouse model of late-onset AD, containing the ApoE ε4 allele; consequently, studies on the role of metals in AD need to take into account the ApoE status of either the human subjects or the mouse models. Furthermore, due to the interaction between different metals such as Cu and Zn, care needs to be taken when conducting both *in vitro* and *in vivo* studies, where the outcomes and interpretations of studies may be confounded by this inter-relationship. Future studies examining the effects of Zn and/or Cu deficiencies and excesses in the different human ApoE alleles of a late-onset model of AD is warranted.

## Conflict of interest statement

The authors declare that the research was conducted in the absence of any commercial or financial relationships that could be construed as a potential conflict of interest.
